# Long term effects of radiation exposure on telomere lengths of leukocytes and its associated biomarkers among atomic-bomb survivors

**DOI:** 10.18632/oncotarget.8801

**Published:** 2016-04-19

**Authors:** Ana Lustig, Ivo Shterev, Susan Geyer, Alvin Shi, Yiqun Hu, Yukari Morishita, Hiroko Nagamura, Keiko Sasaki, Mayumi Maki, Ikue Hayashi, Kyoji Furukawa, Kengo Yoshida, Junko Kajimura, Seishi Kyoizumi, Yoichiro Kusunoki, Waka Ohishi, Kei Nakachi, Nan-ping Weng, Tomonori Hayashi

**Affiliations:** ^1^ Laboratory of Molecular Biology and Immunology, National Institute on Aging, National Institutes of Health, Baltimore, Maryland, USA; ^2^ Duke University, Durham, North Carolina, USA; ^3^ Health Informatics Institute, University of South Florida, Tampa, Florida, USA; ^4^ Department of Radiobiology/Molecular Epidemiology, Radiation Effects Research Foundation (RERF), Hiroshima, Japan; ^5^ Central Research Laboratory, Hiroshima University Faculty of Dentistry, Hiroshima, Japan; ^6^ Department of Statistics, RERF, Hiroshima, Japan; ^7^ Department of Clinical Studies, RERF, Hiroshima, Japan

**Keywords:** ionizing radiation, telomeres, leukocytes, aging, Hiroshima, Gerotarget

## Abstract

Ionizing radiation (IR) is a major source of cellular damage and the immediate cellular response to IR has been well characterized. But the long-term impact of IR on cell function and its relationship with aging are not known. Here, we examined the IR effects on telomere length and other biomarkers 50 to 68 years post-exposure (two time points per person) in survivors of the atomic bombing at Hiroshima during WWII. We found that telomere length of leukocytes was inversely correlated with the dose of IR (*p*=0.008), and this effect was primarily found in survivors who were exposed at younger ages; specifically those <12 years old (*p*=0.0004). Although a dose-related retardation of telomere shortening with age was observed in the cross-sectional data, longitudinal follow-up after 11 years did not show IR exposure-related alteration of the rate of telomere shortening with age. In addition, IR diminished the associations between telomere length and selected aging biomarkers that were observed in survivors with no dose. These included uric acid metabolism, cytokines, and blood T cell counts. These findings showed long-lasting detrimental effects of IR on telomere length of leukocytes in both dose- and age-at-exposure dependent manner, and on alterations of biomarkers with aging.

## INTRODUCTION

Ionizing radiation (IR) causes a wide range of changes in DNA, lipid, and protein leading to profound injury of exposed cells and tissues [1, 2]. The degree of the cellular injuries depends on the dose of radiation, length of exposure, type of exposed cells/tissues, and age of subjects [3–5]. The immediate cellular response to IR is characterized by an appearance of γ-H2AX foci at the DNA damage sites along with the activation and localization of DNA repair and other cellular machineries [6]. When the damages go beyond the normal cellular repair capacity, these radiation-induced lesions either cause cell death or persist and propagate to progeny cells. However, compared to the acute response, the long-term cellular damages of IR are less understood.

Studies of Atomic-bomb (A-bomb) survivors from Hiroshima and Nagasaki, Japan have shown that IR had long-lasting detrimental effects on the immune system. Alterations of the immune system included reduced circulating naive T cells [7] and increased inflammatory cytokine levels such as IL-6, interferon-γ, tumor necrosis factor (TNF)- α, IL-10, and C-reactive protein (CRP) in blood [8, 9].

Telomeres are the end structures of linear chromosomes and play an essential role in protecting the integrity of chromosomes [10]. Due to the inability of conventional DNA polymerase to completely replicate the ends of chromosomes, loss of telomere length occurs after cell division. Cumulative telomere loss from sequential cell divisions over time eventually leads to critically shortened telomeres in the cells, which causes cessation of cell division or apoptosis [11]. Lymphocytes rely on robust cell divisions for their function in combating invading pathogens or tumor cells [12]. Telomere attrition with age in lymphocytes has been reported [13–16] and is considered as an indicator of reduced immune function with age [17]. Several factors such as age, health status, and physiological determinants have been associated with telomere length [16]. Finally, recent studies show that IR exposure causes telomere length shortening *in vitro* [18] and *in vivo* [19]. However, the long-term effect of IR on telomere length and its change with age remains to be determined.

To directly address the long term impact of IR, we conducted a study of 415 selected A-bomb survivors from Hiroshima, Japan with blood collections at a first visit (~55 years after the bombing) and at a second visit on average 11 years later (~66 years after the bombing). Our findings reveal insights into the long-lasting IR effects on telomere length and its change with age.

## RESULTS

### Reduced telomere length of leukocytes was dependent on the dose and the age at IR exposure

**Figure 1:**
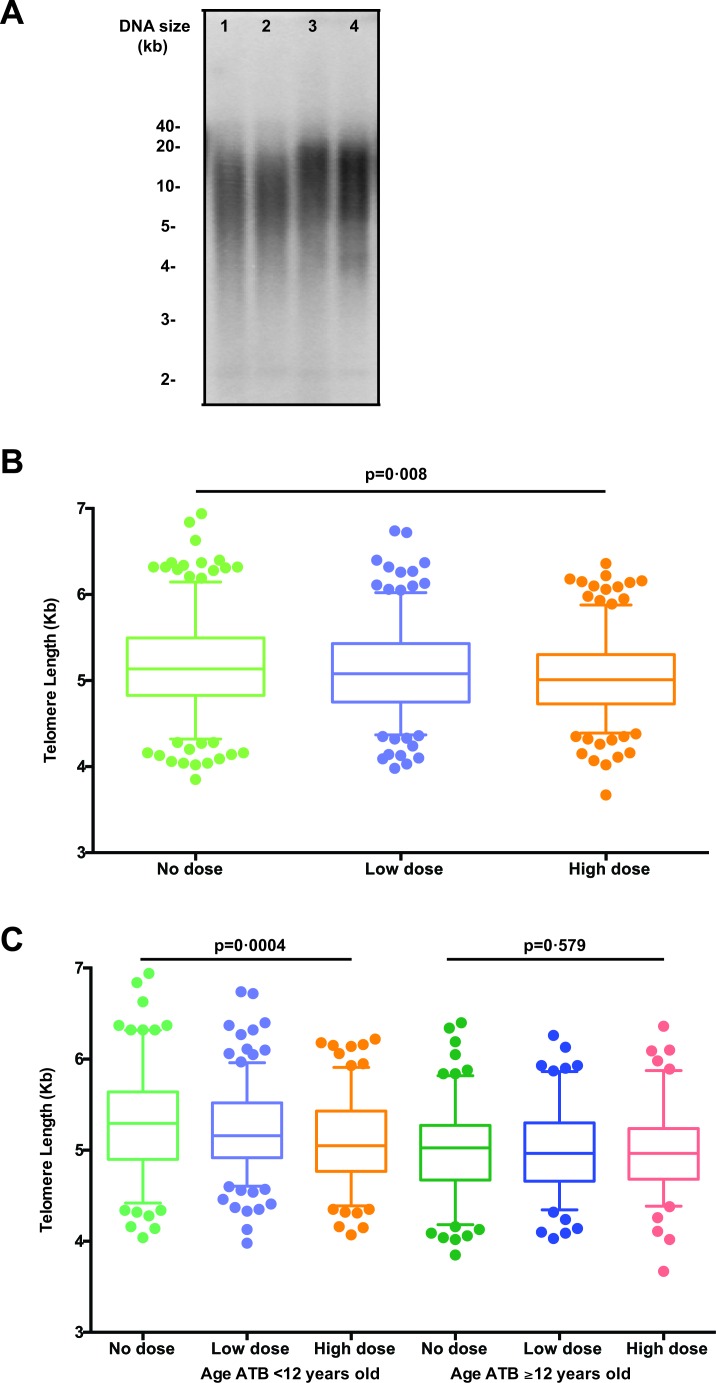
Telomere length for all subjects by dose groups **A.** Representative telomere measurement data by Southern blot. Lanes 1 and 2 are the first and second time points, respectively, from a subject who was aged 16 ATB, and similarly lanes 3 and 4 are from a subject who was aged 2 ATB. **B.** Boxplots of telomere length (averaged across both samples) for all subjects (healthy plus ill) by dose groups. The middle line reflects the median, the box length reflects the interquartile range (Interquartile range, IQR = 75^th^ percentile − 25^th^ percentile), and the whiskers reflect the 5^th^ and 95^th^ percentiles. Points represent specific values in the quantiles beyond the whiskers. Here, telomere lengths appear significantly shorter in those with increased dose groups (*p* for trend = 0.008). **C.** Boxplots of telomere length (averaged across both visits) for all subjects by subject age at the time of the bombing (age ATB < 12 *vs*. ≥ 12 years old) and by dose groups. An interaction effect is shown, where telomere lengths significantly differ between dose groups in those who were age < 12 *vs*. ≥ 12 years old ATB (*p* for trend = 0.0004), but no significant difference was observed across dose groups in those who were ≥ 12 years old ATB (*p* = 0.58).

To examine the effects of IR exposure on telomere length, we used the Southern blot method and analyzed telomere lengths of leukocytes from 415 A-bomb survivors from Hiroshima at the first visit (2000-2002) and second visit after ~11 years (2010-2012) (Table 1) in an observer-blind manner. A representative image of the Southern blot data is shown in Figure 1A.

**Table 1:** Demographics of the study cohort by dose group

Characteristic	No dose	Low dose	High dose	*p* value
	(<5mGy)	(5-700mGy)	(>700mGy)	
Gender	Total	Total	Total	
Female	80	62	78	0.40
Male	77	61	57	
Total	157	123	135	
Age at first visit				
<65 y.o.	78	48	70	0.09
>65 y.o	79	75	65	
Median	65.15	67.95	63.64	0.16
Range	55.1-80.1	55.1-80.0	54.9-79.1	
Age ATB				
<12 y.o.	82	57	82	0.07
≥12 y.o.	75	66	52	
Median	9.6	12.8	7.9	0.66
Range	0.2-23.8	0.2-23.6	0.2-23.6	
Average year span between 2 visits	11	11	10.9	0.66
BM dose (mGy)				
Median		229	1302	
Range		5-694	701-3755	

Based on the dosage of radiation exposure, the survivors were further divided into no (< 5 mGy), low (5-700 mGy), and high (≥ 700 mGy) dose groups. We found a significant trend of reduced telomere length with increased radiation exposure (*p* for trend = 0·08) (Figure 1B). Telomere lengths in the no dose subjects (mean ± SD: 5.16 ± 0.03 Kb) were significantly longer than those of the high dose subjects (5.05 ± 0.03 Kb, Δ = 110 bp, *p* = 0.029), but no significant differences were found between other paired dose groups. It has been reported that subjects who were exposed at younger ages were more prone to IR induced damage than those at older ages [20]. Therefore, we further analyzed telomere length in different age groups based on the age at the time of bombing (ATB). We found that younger survivors (age at ATB < 12 years old) displayed a great radiation dose-dependent reduction of telomere lengths (*p* for trend = 0.0004) but we did not find such a difference in the older age (age at ATB ≥ 12 years old) group (Figure 1C). In the younger age ATB group, the average difference of telomere lengths between no dose subjects (5.30 ± 0.04 Kb) the high dose subjects (5.09 ± 0.04 Kb) was significant (Δ = 210 bp, *p* = 0.010) (Figure 1C). In contrast, telomere lengths of the older survivors (≥ 12 years old ATB) did not differ between any paired exposure groups (Figure 1C). Similar findings were observed when samples from the first and second visits were analyzed separately (Supplemental Figure S1). Collectively, these results demonstrated that IR-induced telomere length attrition in leukocytes was dependent on dose and age at exposure.

### Rate of telomere shortening with age was not affected in A-bomb survivors

**Figure 2:**
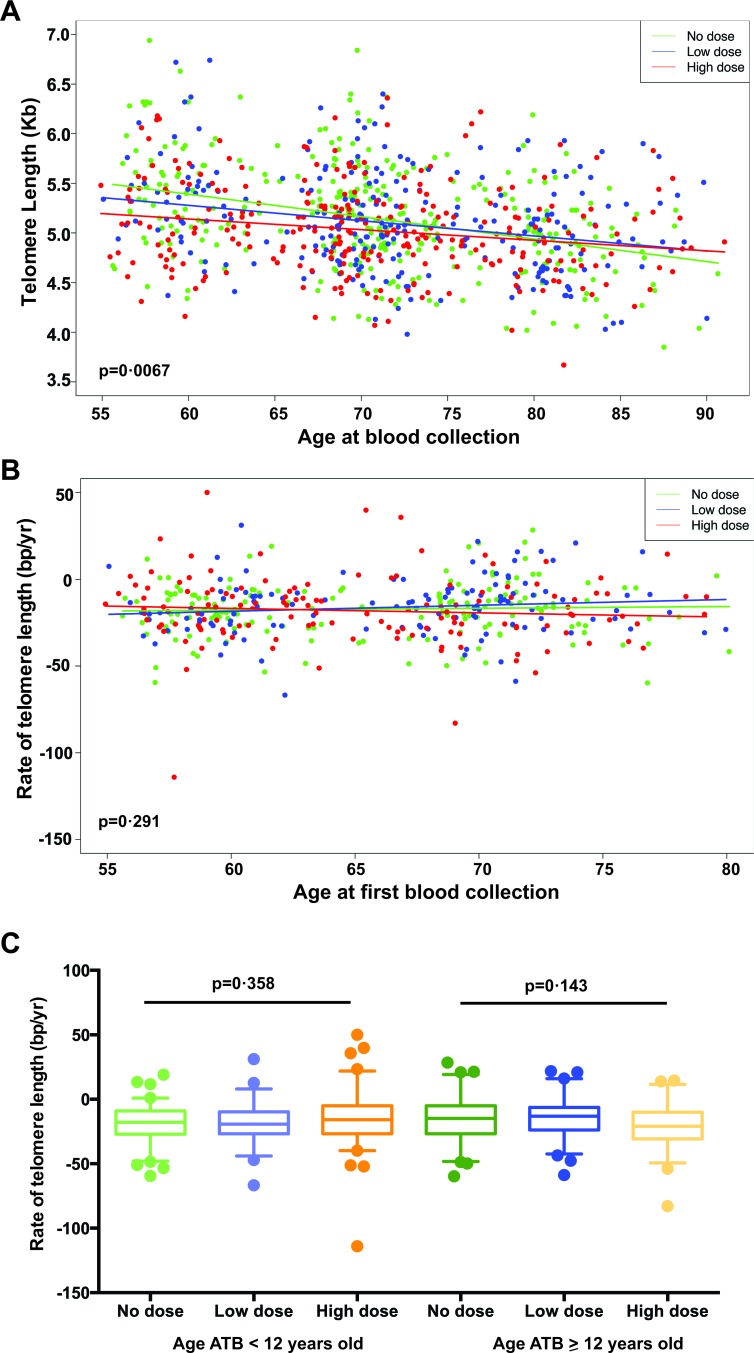
Telomere length change *versus* age in all study subjects **A.** Scatterplot of telomere length *versus* age at blood collection for all subjects (healthy plus ill), and with data for both visits reflected (i.e. 2 points per subject). There is a significant interaction effect with differential relationships between telomere length and age for the different dose groups (*p* = 0.0067). **B.** Scatterplot of the average annual rate of change in telomere length between the first and second visits *versus* the age at the first visit for all subjects. There is no significant interaction effect to differentiate relationships between telomere length change and age by dose groups (*p* = 0.29). **C.** Boxplots of the average annual rate of telomere length change with age for all subjects by subject age at the time of the bombing (age ATB < 12 *vs*. ≥ 12 years old) and by dose groups. No interaction effects were detected for either age < 12 or ≥ 12 years old ATB (*p* for trend = 0.358 and 0.143, respectively).

To determine if IR exposure altered telomere length change with age, we first analyzed the slopes of telomere length in all subjects as a function of age at blood collection using the cross-sectional data and found a significant difference among the dose groups (*p* = 0.0067, Figure 2A). Telomere lengths in the high dose exposure group (−10.5 bp/yr) had a slower loss with age compared to the no dose group (−24.1 bp/yr) (*p* = 0.003) and to the low dose group (−16.9 bp/yr, *p* = 0.05) (Figure 2A). However, these observed slope differences could reflect an IR dose-dependent telomere length attrition in the younger age ATB but not the older age ATB survivors. To address this directly, we compared the rate of telomere length change with age calculated based on the longitudinal data between the first and second visits (~11-year follow-up). All dose groups showed loss of telomere length over time at a similar rate and no significant differences were detected among them from the longitudinal data (Figure 2B). We then examined if the rate of telomere attrition was influenced by the age at exposure and again we did not find differences in the rate of telomere attrition among the three dose groups regardless of the age at IR exposure (Figure 2C). Together, the longitudinal analysis does not show IR exposure altering the rate of telomere length change with age. However, longitudinal analysis with multiple time points and a longer time span may be necessary to determine if some subtle alteration of the rate of telomere length changes might exist.

### Associations of telomere length and selected biomarkers were altered by IR exposure

To further characterize the effects of IR on immune cells, we analyzed the associations of telomere length, epidemiological variables (such as age at blood collection), and 49 biomarkers (Supplemental Table S1) using a multivariate GEE model. To avoid the potential complication of immunological diseases and cancers on these biomarkers, we focused on the comparison of 329 healthy subjects. Significant associations were found between telomere length and various blood biomarkers in all dose groups (Table 2).

**Table 2:** Association of telomere length and biomarkers in healthy subjects

Biomarker	No dose			Low dose			High dose			No-Hi^**^
	Mean	*p*-value^*^	Co-efficient	Mean	*p*-value^*^	Co-efficient	Mean	*p*-value	Co-efficient	*p*-value
Creatinine (mg/dL)	0.7	0.000	−0.8	0.8			0.7	0.003	−0.28	
Uric acid (mg/dL)	5.2	0.000	−0.1	5.1			5.4			0.053
C-reactive protein	0.2			0.2			0.2	0.036	−0.19	
Albumin (mg/dL)	4.5	0.005	0.4	4.4			4.4			0.020
HDL cholesterol (mg/dL)	64.4			59.7			62.9	0.010	0.01	
GCSF (pg/ml)	48.2	0.021	0.0	50.8	0.024	0.0	48.4			
IFNG (pg/ml)	296.9	0.021	0.0	273.1	0.024	0.0	316.5			
IL-7 (pg/ml)	8.9	0.049	0.0	12.7	0.000	0.0	7.8			
IL-1B (pg/ml)	3.4	0.021	0.0	2.6			2.9			
IL-6 (pg/ml)	13.3	0.034	0.0	9.9			20.2			
TNF-α (pg/ml)	175.7	0.034	0.0	95.8			240.5			
IL-10 (pg/ml)	9.9			8.7	0.001	0.0	15.1			
IL-12.p70 (pg/ml)	24.8			37.3	0.006	0.0	26.1			
IL-13 (pg/ml)	24.8			22.9	0.039	0.0	26.1			
% of CD3+ T cells in lymphocyte	67.0	0.000	0.0	66.6			64.9			0.008
% of CD4+ T cells in lymphocyte	45.7	0.021	0.0	44.2	0.011	0.0	44.5			
% of CD4+CD45RA+(naïve) T cells in lymphocyte	17.4	0.000	0.0	15.8	0.052	0.0	16.6			
Ratio of CD4+CD45Ra+ vs. CD4+CD45Ra-	0.7	0.021	0.3	0.6			0.7			
Ratio of CD4/CD8	3.8			3.4	0.052	0.0	3.7			

* *p*-value was derived from a comparison of telomere length and biomarkers using GEE model adjusted with FDR.

** *p*-value was derived from Wilcoxon test between no and high dose groups of the biomarkers.

Intriguingly, among 13 identified associated biomarkers in the no dose group, five were significantly associated with telomere length in the low dose group and only one in the high dose group. This suggests that the relationship of telomere length with these biomarkers was influenced by IR exposure. Of the seven biomarkers that displayed a significant association with telomere length only in the no dose group, six (blood albumin, IL-1β, IL-6, TNF-α, % CD3^+^ T cells, and ratio of CD4CD45RA^+^/CD4CD45RA^−^) were positively and one (blood uric acid) was negatively associated. Five biomarkers (GCSF, IFN-γ, IL-7, % of CD4^+^ T cells and % of CD4^+^CD45RA^+^ T cells) showed significant positive correlations with telomere length found in both no and low dose groups. One negatively correlated biomarker, creatinine, was significant in both no and high dose groups (Table 2). Four biomarkers (IL-10, IL-12p70, IL-13 and ratio of CD4/CD8) exhibited a positive correlation with telomere length only in the low dose group while two biomarkers (negatively correlated C-reactive protein and positively correlated HDL) were found only in the high dose group. To determine if the loss of association with telomere length is due to the alteration of these biomarkers after IR exposure, we directly compared these biomarkers between the no and high dose groups and found significant differences in the levels of uric acid, albumin, and % CD3^+^T cells (Table 2). We also compared the temporal changes of telomere length and these biomarkers but did not find any significant association. Collectively, we have identified a set of biomarkers that were correlated with telomere length and determined that IR exposure altered these biomarker levels, resulting in the loss of their correlation with telomere length.

### Influence of diseases on telomere length appeared to be dose related

**Figure 3:**
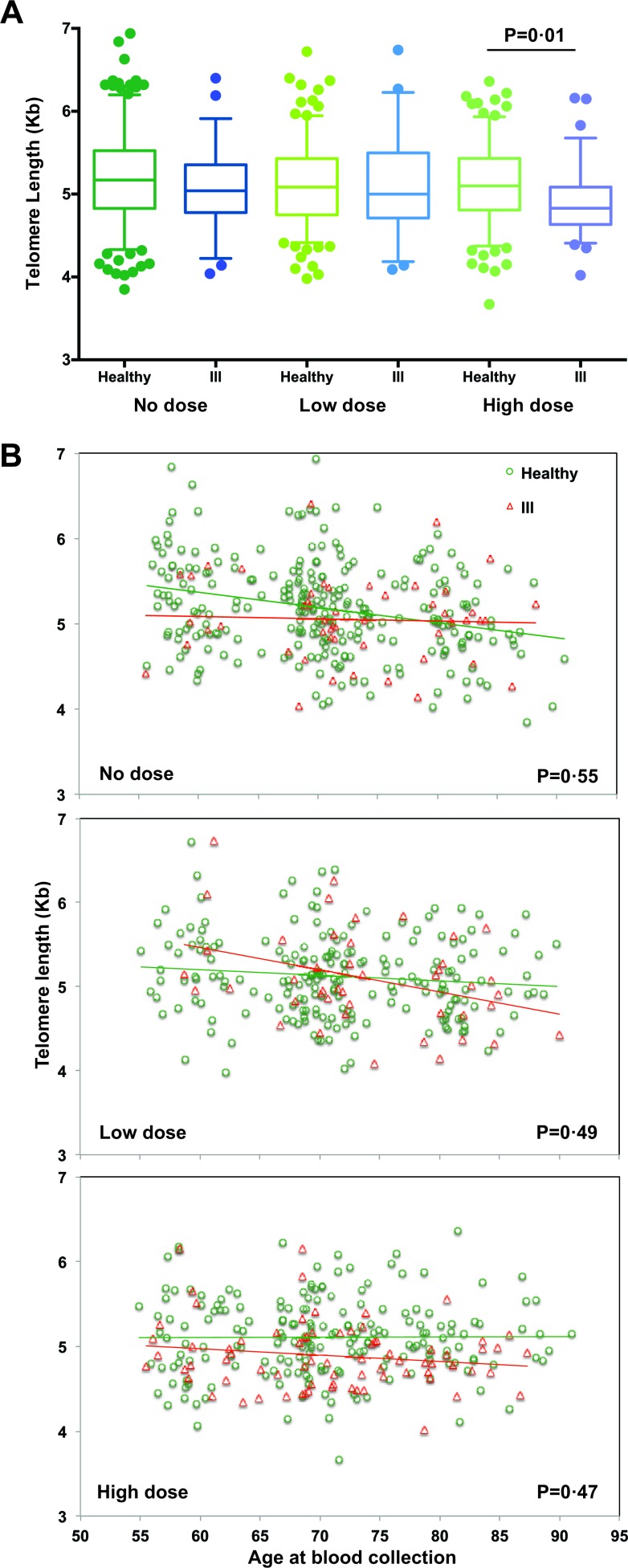
Comparison of telomere length and age at blood collection between healthy and ill subjects **A.** Boxplots of telomere length for healthy *vs*. ill subjects separated by dose groups. The middle line reflects the median, the box length reflects the interquartile range (IQR = 75^th^ percentile − 25^th^ percentile), and the whiskers reflect the 5^th^ and 95^th^ percentiles. Points plotted beyond those lines reflect outliers. Here, a significant difference in the distribution of telomere lengths was only observed between healthy and ill subjects in those who were in the high dose group (*p* = 0.01). **B.** Scatterplots of telomere length *versus* age at blood collection for both visits by healthy *vs*. ill subjects and separated for each dose group. No significant difference in telomere length change with age between healthy and ill subjects was observed regardless of dose groups, based on the generalized estimating equation (GEE) models.

Among the 415 study subjects who were healthy at the first visit, 329 subjects remained healthy at the second visit but 86 subjects were diagnosed with either immune-related diseases after the first visit or cancers within 5 years prior to the second visit (demographic information in Supplemental Tables S2-3 and leading disease list in Supplemental Table S4) and were assigned to the ill group. To determine whether these diseases impact telomere length, we compared healthy and ill subjects in each dose group in the cross-sectional analysis and found that telomere lengths of healthy subjects were significantly longer than those of ill subjects only in the high dose group (*p* = 0.01) but not in the no dose and low dose groups (Figure 3A, Supplemental Figures S2-S3). We did not find significant longitudinal differences in telomere length changes with age between healthy and ill subjects in all three dose groups (Figure 3B and Supplemental Figure S4).

## DISCUSSION

We aimed to determine the long-term impact of IR exposure on telomere length, its change with age, and biomarkers in A-bomb survivors, and found that telomere lengths of leukocytes displayed IR dose-dependent loss, and this dose effect on telomere length was found in subjects who were exposed to IR at younger ages, specifically < 12 years old. These findings demonstrate the long lasting detrimental effect of IR on telomere lengths of leukocytes even 50 to 68 years after the exposure. However, we did not find evidence of IR exposure altering the rate of telomere length attrition with age at blood collection from the longitudinal analysis. In addition, alterations of selected biomarkers that were positively and negatively associated with telomere length were also observed. But the nature of this irreparable damage and its underlying mechanisms of telomere length changes that persist in the progeny cells over half of a century later remain to be elucidated.

The A-bomb survivors have an increased risk of solid cancer incidence for those who received IR exposure at a young age [21], which suggests that the impact of IR-induced cellular damage may be more severe in young than in old subjects. In agreement with this finding, we found significantly shortened telomeres in the leukocytes of the survivors exposed at younger ages. Based on the rate of telomere attrition in the no dose group (−16 bp/yr), the average telomere loss of 210 base pairs more in high dose than in no dose in the age ATB < 12 years old group can be estimated to be approximately 13 years of telomere shortening. As the short telomere lengths of leukocytes were observed many decades after the IR exposure, the damage most likely was derived from initial lesions in the hematopoietic stem cells that transmit over the progeny of differentiated leukocytes. Although it is not entirely clear what accounts for the different IR sensitivity of stem cells between young and old subjects, a recent study of the developing mouse intestine response to IR has pointed to distinct IR-induced apoptosis responses between neonates and adults [22]. Neonate intestinal crypts are much more resistant to IR-induced apoptosis in terms of time and magnitude of apoptosis than adult intestinal crypts. The surviving young stem cells then carry some permanent DNA scars that pass onto their progenitor cells. This may explain why the IR impact on telomere length is still visible over a half century later.

Does IR exposure result in a long lasting alteration or impairment of the cellular repair machinery? Our cross-sectional analysis showed a radiation dose-dependent reduction in the steepness of the slope of telomere shortening with age at blood collection (Figure 2A), which could reflect the differential impact of IR exposure on telomere length between younger and older subjects. Furthermore, the longitudinal data from the ~11-year follow-up showed no substantial differences in the rate of telomere length change with age at blood collection among different IR dose groups (Figure 2B). Collectively, this suggests that there is no obvious IR exposure-induced long-term impairment on telomere maintenance with age. It is warranted that further studies including the continued follow-up of study subjects with multiple time point samples will be necessary to determine if there is any difference between the subjects in the high and no dose groups in telomere length attrition with age.

Previous studies showed that short telomeres of leukocytes correlated with some physiological/pathological conditions and biomarkers [16, 23] and IR exposure causes activation of the inflammasome pathway in immune cells resulting in altered cytokine expression [24]. If the “normal” associations between telomere length and biomarkers that were seen in the no dose group were lost in the exposed groups, this suggests that IR altered physiological conditions related to those biomarkers, possibly generating a detrimental impact on the telomere lengths of leukocytes. Indeed, a significant dose-dependent increase of blood uric acid and decrease of blood albumin were observed, along with the loss of the negative and positive associations of telomere length with uric acid and albumin, respectively. This notion was further supported by our findings that healthy subjects have significantly longer telomeres than ill subjects in the high dose group. Based on these findings, we think that the associations between biomarkers and telomere length of leukocytes reflect a combinatorial effect from different types of cells and their unique manifestations in response to telomere length. Short telomeres in lymphocytes are likely associated with reduced proliferation and functions (negative effect in general) whereas short telomeres in myeloid-derived cells could lead to reduced expression of some inflammatory cytokines (may be a positive effect with aging). Future studies will be required in order to identify the precise long-lasting lesions resulting from IR exposure and to elucidate how such lesions influence telomere length and its maintenance with age.

## MATERIALS AND METHODS

### Study populations

415 subjects were selected from A-bomb survivors of the Adult Health Study (AHS) at the Radiation Effects Research Foundation (RERF) of Hiroshima, using a stratified random sampling method for gender, radiation dose, and age categories. The demographic information regarding radiation dose, age at the bombing, age at blood collection, and gender distributions of all subjects is shown in Table 1. All subjects were healthy at the first visit as those received radiotherapy, or were undergoing chemotherapy, steroid therapy, or interferon therapy at the time of blood collection, and had a cancer history within 5 years before the first blood collection were excluded. 329 subjects who were healthy at the second visit were considered as the “healthy” group whereas 86 subjects who were diagnosed with either selected immune-related diseases after the first visit or various types of cancers within 5 years of the second visit as the “ill” group (Supplemental Tables S2-3). The diseases that were frequently diagnosed in the 86 subjects are listed in Supplemental Table S4.

Written informed consent was obtained from all subjects prior to this study, which was conducted in accordance with the Ethical Guidelines for Epidemiological Research established by the Ministry of Education, Culture, Sports, Science and Technology, and the Ministry of Health, Labor and Welfare of Japan, the Declaration of Helsinki, and was approved by the RERF ethical committee. The blood samples were collected in 2000-2002 (first visit) and in 2010-2012 (second visit), and standard blood chemistry analysis was done at RERF.

### Radiation dose

The radiation dose is the γ dose plus 10 times the neutron dose, using the bone marrow doses calculated by the modified dosimetry system (DS02) [21, 25]. DS02 used more accurate digital terrain elevation data on a 10-m horizontal grid available from the Geospatial Information Authority of Japan rather than only for pre-selected groups based on arbitrary criteria. The study subjects were divided into three groups according to their radiation dose levels: those exposed to radiation doses < 5 mGy were referred to as “no dose”, those exposed to radiation doses of 5-700 mGy as low, and ≥ 700 mGy as high dose group.

### Measurement of telomere length

Telomere length was measured by Southern blot previously described [26] and was conducted at RERF. In brief, genomic DNA was isolated from leukocytes using a DNA isolation kit (Qiagen) and digested with *Hinf*I and *Rsa*I (NEB). Digested DNA was separated on a 0.6% agarose gel by electrophoresis. The hybridization was performed with a [^32^P] end-labeled oligonucleotide (TTAGGG)_4_ probe, at 45°C overnight. The images were acquired by a phosphor imager (Typhoon 9410; GE Biosciences). Mean telomere length (terminal restriction fragment, TRF) was calculated at the NIA in an observer blind manner.

### Multiplex cytokine/chemokine/growth factor analysis

Cytokine/chemokine/growth factor profiling of plasma samples was performed using a Luminex bead-based Bio-Plex Pro Human Cytokine, Chemokine, and Growth Factor 27-plex assay (Bio-Rad, Hercules, CA, USA) according to the manufacturer's protocol at RERF. Each sample was assayed in singlet and all samples were run on lot-matched assay kits. Final observed pg/mL of each analyte in the assay was quantified using a 5PL curve fit of assay kit standards using Bioplex Manager Software.

### Statistical analysis

The Wilcoxon rank-sum test was used to compare continuous measures (e.g. telomere length) between two groups (healthy *versus* ill, female *versus* male, no dose *vs*. high dose, etc.) [27]. The Kruskal-Wallis rank-sum test[28] was used to assess differential distributions and compare continuous measures across the three dose groups. The Jonckheere-Terpstra (JT) test was used to test for ordered differences in dose groups [29]. To accommodate for potential intra-subject correlation, analyses including both the first and second visit samples were done using generalized estimating equation (GEE) models to assess the influence and relationships with various variables such as age at blood collection and the biomarkers (measured with blood samples collected at first and second visits) [30, 31]. Relationships of these variables with the average annual rate change in subjects were evaluated using generalized linear regression models. These GEE and GLM models were used to assess relationships with all variables in the univariate setting; significant variables were further evaluated in relation to the telomere measures in the multivariable setting. In addition, interactions were also tested in the context of these GEE and GLM models, as appropriate for the dependent variable. Asymptotic p-values were computed for all tests and comparisons; to adjust for the multiple comparisons, p-values were also adjusted per the Benjamini-Hochberg FDR correction for the univariate analyses [32, 33]. All analyses were done using R statistical program (version 3.1.1) ^29^ for Linux, including the R packages geepack (version 1.2-0) ^26^ and clinfun (version 1.0.10).

## SUPPLEMENTAL MATERIAL



## Abbreviations

IR: ionizing radiation
ATB: at time of bombing
