# Supplementary material for: Long term effects of radiation exposure on telomere lengths of leukocytes and its associated biomarkers among atomic-bomb survivors
Source: Oncotarget. 2016 Apr 19;7(26):38988–98. doi: 10.18632/oncotarget.8801 (PMC5129908; doi:10.18632/oncotarget.8801)
Supplement: Supplementary file 1 [file oncotarget-07-38988-s001.pdf]

Long term effects of radiation exposure on telomere lengths of leukocytes and its associated biomarkers among atomic-bomb survivors

Supplemental Material

Study subject selection and exclusion

Supplemental Figure S1: Telomere length by dose exposure and age at time of atomic bomb grouped by each visit.

Supplemental Figure S2: Telomere length by dose and age ATB for healthy and ill subjects.

Supplemental Figure S3: Telomere length versus age at first blood collection, by dose groups comparing healthy to ill subjects.

Supplemental Figure S4: Telomere length change versus age at blood collection by dose groups, comparing healthy to ill subjects.

Table S1 List of biomarkers

Table S2 Demographics of healthy vs. ill survivors, grouped by exposure dose

Table S3 Demographics comparing healthy to ill survivors in each dose group

Table S4 Leading immune-related diseases and cancers in ill subjects

Study subject selection and exclusion

Initially, 452 subjects were selected from 2,300 A-bomb survivors from Hiroshima participating in the Adult Health Study (AHS) at the Radiation Effects Research Foundation (RERF), using a stratified random sampling method for the gender, radiation dose, and age category. Their blood samples were collected in 2000-2002 (first visit) and in 2010-2012 (second visit) and the mean time-interval between the first and second visit was 11 years (range: 8.7 to 13.1 years). Thirty seven subjects from those 452 were excluded for the reason that they received radiotherapy, or were undergoing chemotherapy, steroid therapy, or interferon therapy at the time of blood collection, and had a cancer history within 5 years before the first blood collection. The final 415 subjects were used in this study.

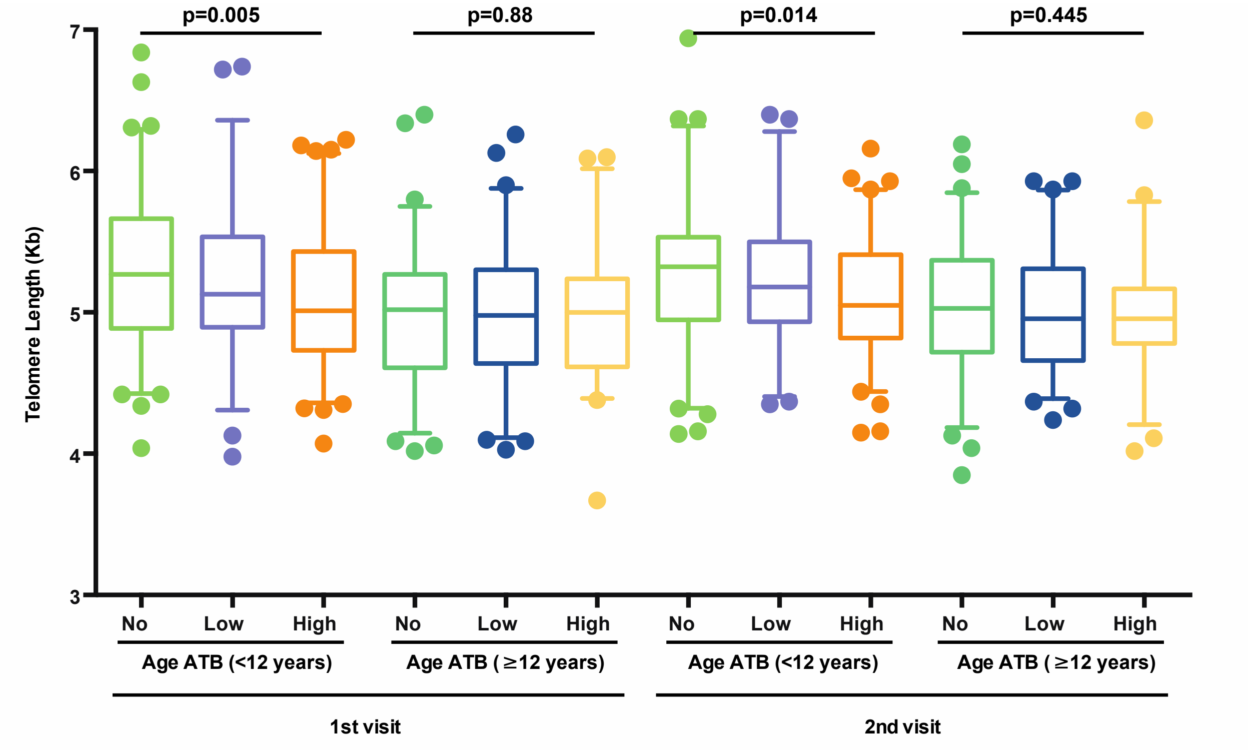

Supplemental Figure S1: Telomere length by dose and age ATB at the first and second visits. Boxplots of telomere length for all subjects (healthy plus ill) for different dose groups by age ATB and separated by the first and second visits. The middle line reflects the median, the box length reflects the interquartile range (IQR = 75th percentile – 25th percentile), and the whiskers reflect the 5th and 95th percentiles. Points represent specific values in the quantiles beyond the whiskers. Here, only the subjects younger than 12 years old ATB displayed differential telomere lengths according to radiation dose, where there was a significant shortening trend of telomere length with increased dose for both the first and second visit blood samples (p for trend=0.005 and 0.014, respectively).

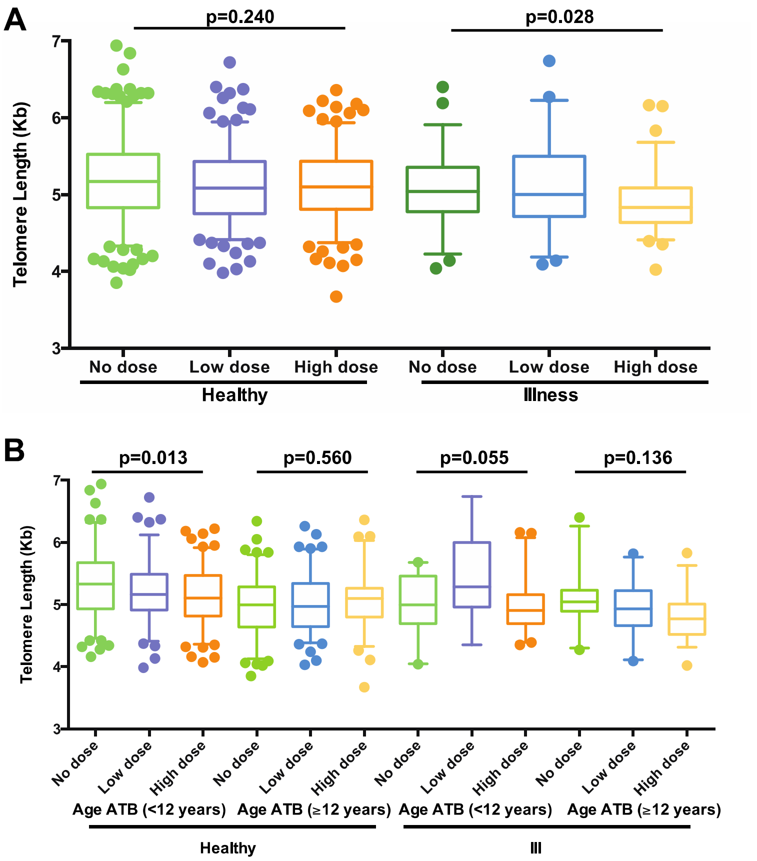

Supplemental Figure S2: Telomere length by dose and age ATB for healthy and ill subjects. A) Boxplots of telomere length for all subjects (healthy plus ill) for different dose groups separated by whether or not the subject had an underlying illness (healthy vs. ill). The middle line reflects the median, the box length reflects the interquartile range (IQR = 75th percentile – 25th percentile), and the whiskers reflect the 5th and 95th percentiles. Points represent specific values in the quantiles beyond the whiskers. Here, exposure dose did not appear significantly influential on telomere length distributions in healthy subjects; however, there appeared to be a trend toward differential telomere lengths, particularly in high dose ill subjects (p for trend=0.028). B) Boxplots of telomere lengths for all subjects for different dose groups by age ATB and compared healthy to ill subjects. Here, we see a significantly decreasing trend of telomere lengths with increased dose in healthy subjects who were younger than 12 years old ATB (p for trend=0.013). The ill subjects showed a similar trend although it did not reach a statistically significant level (p=0.14) due to the small number of subjects.

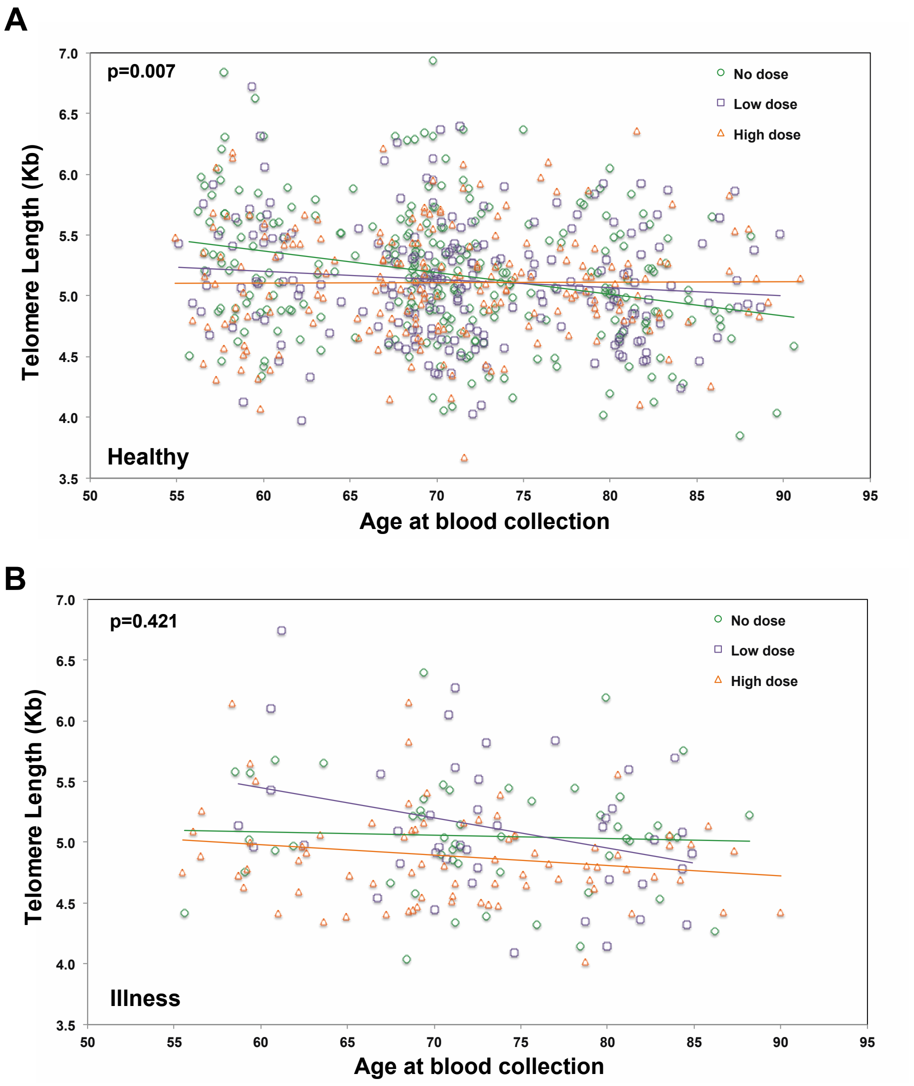

Supplemental Figure S3: Telomere length versus age at blood collection by dose groups comparing healthy to ill subjects. A) Scatterplot of telomere length versus age at blood collection by dose groups for healthy subjects, with data for both visits reflected (i.e. 2 points per subject). Relationship between telomere length and age at blood collection significantly differs according to dose groups in healthy subjects (p for trend=0.007). B) Scatterplot of telomere length versus age at blood collection by dose groups for ill subjects, with data for both visits reflected (i.e. 2 points per subject). There was no significant interaction effect to differentiate the relationship between telomere length and age at blood collection by dose groups.

A

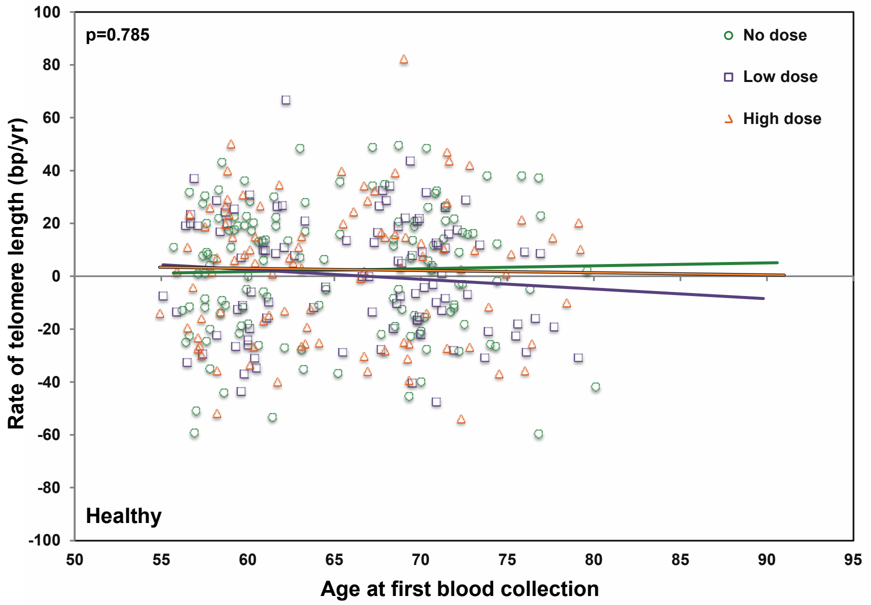

B

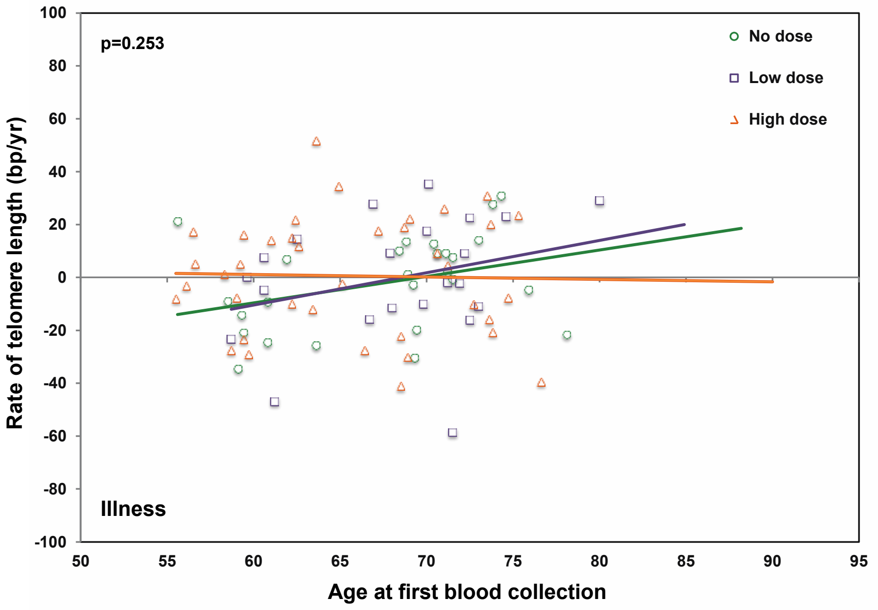

Supplemental Figure S4: Telomere length change versus age at first blood collection by dose groups, comparing healthy to ill subjects. A) Scatterplot of the average annual rate of change in telomere lengths between the first and second visits versus the age at the first visit for healthy subjects. There was no significant interaction effect to differentiate relationships between telomere length change and age at first blood collection by dose groups in healthy subjects (p=0.79). B) Scatterplot of the average annual rate of change in telomere lengths between the first and second visits versus the age at first blood collection for ill subjects. There was no significant interaction effect to differentiate relationships between telomere length change and age by dose groups in ill subjects.

Table S1: List of biomarkers

Name	Unit
Body mass index	kg/m2
Systolic blood pressure	mmHg
Diastoric blood pressure	mmHg
Hemoglobin	g/dl
White Blood Cell	x100 cells/ul
Neutrophils	%
Lymphocytes	%
Monocytes	%
Eosinophils	%
Basophils	%
Albumin	g/dL
Hemoglobin A1C	%
Total cholesterol	mg/dL
HDL cholesterol	mg/dL
LDL cholesterol	mg/dL
Trigyceride	mg/dL
Creatinine	mg/dL
Uric acid	mg/dL
C-reactive protein	mg/dL
CD3+ T cells in lymphocyte	%
CD4+ T cells in lymphocyte	%
CD8+ T cells in lymphocyte	%
CD4+CD45RA+(naïve) T cells in lymphocyte	%
CD4+CD45RA-(memory) T cells in lymphocyte	%
CD4/CD8 T cells	%
CD4+CD45Ra+ vs. CD4+CD45Ra-	ratio
IL-1b	pg/ml
IL-2	pg/ml
IL-4	pg/ml
IL-5	pg/ml
IL-6	pg/ml
IL-7	pg/ml
IL-8	pg/ml
I-L9	pg/ml
IL-10	pg/ml
IL-12p70	pg/ml
IL-13	pg/ml
IL-15	pg/ml
IL-17	pg/ml
Eotaxin	pg/ml
GCSF	pg/ml
IFN-γ	pg/ml
IP10	pg/ml
MCP1	pg/ml
MIP1b	pg/ml
PDGFbb	pg/ml
TNF	pg/ml
VEGF	pg/ml
RANTES	pg/ml

Table S2: Demographics of the study subjects grouped according to exposure dose and health status

	Healthy subject				Ill subject			
Characteristic	No exposure (<5 mGy)	Low dose (5-700 mGy)	High dose (700+ mGy)	p-value	No exposure (<5 mGy)	Low dose (5-700 mGy)	High dose (700+ mGy)	p-value
Gender								
Female	70	54	55	0.87	10	8	23	0.12
Male	61	47	42		16	14	15	
Total	131	101	97		26	22	38	
Age at early sample								
<65 y.o.	69	42	51	0.19	9	6	19	0.20
>=65 y.o.	62	59	46		17	16	19	
Median	63.3	67.7	63.4	0.29	69.2	69.9	65	0.25
Range	55.7 to 80.1	55.1 to 79.1	54.9 to 79.2		55.6 to 78.1	58.7 to 80.0	55.5 to 76.6	
Age ATB								
<12 y.o.		49	62	0.032		8	21	0.19
>=12 y.o.		52	35			14	17	
Median		12.4	7.8	0.13		14	9.7	0.14
Range		0.2 to 23.5	0.2 to 23.6			3.9 to 23.6	0.8 to 20.5	
Average year span between 2 visits								
BM dose (mGy)								
Median		251	1295			186	1351	
Range		5 to 694	701 to 3755			5 to 683	701 to 3213	

Table S3: Demographics of healthy vs ill A-bomb survivors grouped according to exposure dose

	No dose (<5mGy)			Low dose (5-700mGy)			High dose (>700mGy)		
Characteristic	Healthy	Ill	p	Healthy	Ill	p	Healthy	Ill	p
	126	31		89	34		89	46	
Gender									
female	67	13	0.3571	50	12	0.06	53	25	0.69
male	59	18		39	22		36	21	
Age ATB									
< 12 y.o.	70	12	0.1385	44	13	0.36	57	25	0.36
>= 12 y.o.	56	19		45	21		32	21	
median	7.7	13.9	0.011	12.4	13.7	0.1	7.6	11	0.26
range	0.2 to 23.8	0.3 to 22		0.2 to 23.5	2.4 to 23.6		0.2 to 23.6	0.8 to 20.7	
Age at early sample									
< 65 y.o.	67	11	0.12	38	10	0.25	48	22	0.62
>= 65 y.o.	59	20		51	24		41	24	
median	63.26	69.3	0.017	67.6	69.59	0.069	63.31	65.76	0.332
range	55.7 to 80.1	55.6 to 78.1		55.1 to 79.1	57.4 to 80		54.9 to 79.2	55.5 to 76.6	
Average year span between 2 visits	10.8	10.5	0.84	11.0	10.8	0.98	10.4	10.4	0.84
BM Dose (mGy)									
median				229	221	0.28	1261	1351	0.88
range				5 to 694	5 to 683		701 to 3755	701 to 3213	

Table S4: Leading immune-related diseases and cancers in ill subjects*

Immune-related diseases	Subject#
Chronic viral hepatitis	15
Malignant neoplasms, stated or presumed to be primary, of lymphoid, haematopoietic and related tissue	2
Neoplasms, polycythaemia vera	2
Other aplastic anaemias	1
Agranulocytosis	2
Other diseases of blood and blood-forming organs	1
Ulcerative colitis	1
Primary biliary cirrhosis	1
Other rheumatoid arthritis	2
Systemic connective tissue disorders	1
Unspecified anaemia (under treatment with erythropoietin)	1
Chronic respiratory diseases (under treatment with steroid drug)	3
Cancers	
Malignant neoplasms of digestive organs	17
Malignant neoplasms of respiratory and intrathoracic organs	5
Malignant neoplasms of mesothelial and soft tissue	1
Malignant neoplasm of breast	5
Malignant neoplasms of female genital organs	4
Malignant neoplasms of male genital organs	10
Malignant neoplasms of urinary tract	7
Malignant neoplasms of thyroid and other endocrine glands	4
Malignant neoplasms of ill-defined, secondary and unspecified sites	1

* Seven subjects had two diseases (1 subject had IMM-related disease and cancer, 1 subject two IMM-related diseases, and 5 subjects two cancers)
